# Proinflammatory Cytokines and Bile Acids Upregulate ΔNp73 Protein, an Inhibitor of p53 and p73 Tumor Suppressors

**DOI:** 10.1371/journal.pone.0064306

**Published:** 2013-05-22

**Authors:** Elena Zaika, Vikas Bhardwaj, Jinxiong Wei, Mary Kay Washington, Rhonda Souza, Wael El-Rifai, Alexander Zaika

**Affiliations:** 1 Department of Surgery, Vanderbilt University Medical Center and Vanderbilt-Ingram Cancer Center, Nashville, Tennessee, United States of America; 2 Department of Pathology, Vanderbilt University Medical Center, Nashville, Tennessee, United States of America; 3 Department of Cancer Biology, Vanderbilt University Medical Center, Nashville, Tennessee, United States of America; 4 Departments of Medicine, VA North Texas Health Care System and the University of Texas Southwestern Medical School, Dallas, Texas, United States of America; Northwestern University Feinberg School of Medicine, United States of America

## Abstract

Gastroesophageal reflux disease (GERD) is the main etiological factor behind the recent rapid increase in the incidence of esophageal adenocarcinoma. During reflux, esophageal cells are exposed to bile at low pH resulting in cellular damage and inflammation, which are known to facilitate cancer development. In this study, we investigated the regulation of p73 isoform, ΔNp73α, in the reflux condition. Previous studies have reported that ΔNp73 exhibits anti-apoptotic and oncogenic properties through inhibition of p53 and p73 proteins. We found that direct exposure of esophageal cells to bile acids in an acidic environment alters the phosphorylation of ΔNp73, its subcellular localization and increases ΔNp73 protein levels. Upregulation of ΔNp73 was also observed in esophageal tissues collected from patients with GERD and Barrett’s metaplasia, a precancerous lesion in the esophagus associated with gastric reflux. c-Abl, p38 MAPK, and IKK protein kinases were identified to interact in the regulation of ΔNp73. Their inhibition with chemotherapeutic agents and siRNA suppresses ΔNp73. We also found that pro-inflammatory cytokines, IL-1β and TNFα, are potent inducers of ΔNp73α, which further enhance the bile acids/acid effect. Combined, our studies provide evidence that gastroesophageal reflux alters the regulation of oncogenic ΔNp73 isoform that may facilitate tumorigenic transformation of esophageal metaplastic epithelium.

## Introduction

Esophageal adenocarcinoma (EA) is one of the fastest rising tumors in the United States and Western World, accounting for a 6-fold increase in incidence in the past three decades [1]. The major risk factor for this cancer is gastroesophageal reflux disease (GERD), which affects approximately 10–20% of the population in the US and Western World [2]. Because of the disease, esophageal cells are exposed to acidic gastric juice frequently mixed with duodenal bile acids (BA). The refluxate causes strong cellular and DNA damages and induces inflammation that, in turn, exacerbates the mucosal injury [3]. Constant exposure to pro-inflammatory cytokines, such as IL-1β and TNFα, has been shown to be an important contributing factor to GERD-associated tumorigenesis [4]. The reflux-induced damage may lead to Barrett’s metaplasia (BE), in which normal epithelium is replaced by Barrett’s intestinal type epithelium. In some patients, this precancerous lesion may progress to esophageal dysplasia and adenocarcinoma, although little is currently known about specific mechanisms causing tumorigenic transformation of BE epithelium.

p53 is an important regulator of DNA damage response and a key tumor suppressor. Its inactivation predisposes Barrett’s epithelial cells to the genomic instability and facilitates progression to cancer [5]. p53 is also the founding member of a family of proteins, which includes two additional members, p63 and p73. These proteins have significant functional and structural similarities to p53, although certain specific differences exist in their regulation [6]. Previous studies have found that p63 is downregulated following exposure to bile acids/acid, while p73 is induced and plays an important role in the regulation of DNA damage repair in esophageal cells [7], [8]. However, function of p73 is isoform-specific. The TP73 gene encapsulates “two-in-one” (tumor suppressor and oncogene) activities. N-terminally truncated p73 isoform, ΔNp73α, which lacks the transactivation domain, functions as a dominant-negative and oncogenic protein [9]. It interacts with p53 and p73 proteins and inhibits their transcriptional and pro-apoptotic activities. When expressed, ΔNp73 exacerbates DNA damage induced by BA/A, immortalizes murine cells and induces their anchorage-independent growth [8], [10]. It also cooperates with other cellular oncogenes in cellular transformation and tumor development in mice [11]–[13]. ΔNp73 is frequently over-expressed in human tumors including EA, and its level significantly correlates with poor patient survival in a number of human malignancies [9]. However, regulation of the ΔNp73 protein remains largely unknown.

Here we investigated the regulation of the ΔNp73 protein in conditions of gastroesophageal reflux.

## Materials and Methods

### Cells Cultures, Transfections, Treatment

Human telomerase-immortalized CP-A (ATCC) and BAR-T1 (generated in Dr. Souza laboratory [14]) cell lines, isolated from human Barrett’s metaplasia, were cultured in keratinocyte-SFM (KSFM) medium supplemented with 40 µg/ml bovine pituitary extract and 1.0 ng/ml epidermal growth factor (Life Technologies). Human p53-null esophageal adenocarcinoma cell line SK-GT-4 [15] and human gastric cancer cell line AGS (ATCC) were maintained in DMEM and F12 media, respectively, both supplemented with 10% FBS, 100 u/ml penicillin and 100 µg/ml streptomycin (Life Technologies).

For the generation of cell lines stably expressing human ΔNp73α protein, cells were transfected with vector FLAG-ΔNp73α-pcDNA3 and selected with G418 (Mediatech). The following mammalian expression vectors were used: c-Abl (P242E/P249E)-pcDNA3 (gift from Dr. J. Wang, UC San Diego), IKKα (S176E/S180E)-pCMV and IKKβ (S177E/S181E)-pCMV2 (kind gift from Dr. D. Ballard, Vanderbilt University), p38-pMT3 (Addgene), and MKK6 (S207E/T211E)-pcDNA3 (Addgene). siRNAs against IKKα, IKKβ and p38 were from Cell Signaling, c-Abl and control siRNAs were from Life Technologies. Cells were transfected with Lipofectamine 2000 (Life Technologies) following the manufacturer’s protocols.

Cells were treated with bile acids cocktail (BA) consisting of a 20 µM equimolar mixture of glycocholic acid, taurocholic acid, glycodeoxycholic acid, glycochenodeoxycholic acid, and deoxycholic acid sodium salts (all reagents from Sigma-Aldrich); total BA concentration was 100 µM. For cell treatment, BA cocktail was diluted in DMEM, pH 4.0 (BA/A); pH was adjusted with HCl. Human cytokines, TNFα and IL-1β were purchased from PeproTech. Cell survival was analyzed using MTT analysis as previously described [16].

Specific kinase inhibitors of c-Abl (Imatinib; Euroasian Chemicals PVT.LTD), IKK (Bay 11–7085; EMD Millipore), p38 MAPK (SB203580; Promega), and Aurora A (MLN8237; Selleckchem) were added to growth medium at the indicated concentrations one hour before and immediately after treatment with BA/A.

### Antibody and Immunoprecipitation

Antibodies for the following proteins were used: ΔNp73 (N-16), c-Abl (K-12) and phospho-p73 (Tyr99) from Santa Cruz Biotechnology; p73 from Bethyl Laboratories, phospho-c-Abl (Tyr412), phospho-serine (PSR-45) and FLAG-tag (M2) from Sigma-Aldrich; Phospho-IKKα/β (16A6), IKKα (#2682), IKK β (L570), phospho-Aurora A (Thr288) (C39D8), Aurora A (1G4), DYKDDDDK tag, β-actin (13E5), phospho-p38 MAPK (Thr180/Tyr182), and p38 MAPK (#9212) from Cell Signaling, p73 (Ab2) and phosphotyrosine (4G10) from Millipore. Immunoprecipitation was performed with ΔNp73 (N-16) antibody or Anti-FLAG M2 affinity gel (Sigma-Aldrich) following manufacturer’s protocol.

### Immunohistochemistry and Immunofluorescence

After the Institutional Review Board’s approval, 16 specimens from patients with Barrett’s esophagus, 7 normal gastric and 5 normal esophageal specimens were collected at Vanderbilt University Medical Center, histologically verified and used for immunohistochemical staining for ΔNp73. Immunohistochemical staining of BE esophageal tissues was performed using ΔNp73-specific antibody (Imgenex) at 1∶200 dilution as previously described [17].

For indirect immunofluorescence staining, cells were grown to 50% confluency on chamber slides, treated with BA/A for 30 min and 7 hours after treatment were fixed in 1∶1 solution of methanol:acetone. The fixed cells were treated with 10% goat serum (Life Technologies) and incubated with the FLAG-tag(M2) antibody at 4°C for 16–18 hours and then with AlexaFluor 568-conjugated secondary antibody (Life Technologies) for 45 min at room temperature. Slides were photographed using Olympus BX41 fluorescent microscope (Olympus Co.).

### Ethics Statement

The use of all human pathology specimens for research was approved by the Institutional Review Board (IRB) of Vanderbilt University Medical Center (Vanderbilt University, Nashville TN, USA). Since only de-identified tissues were included in this retrospective study, the IRB has waived requirements for informed consent.

### Real-time PCR

RNA was isolated using the RNeasy kit (Qiagen). Total RNA (1 µg) was reverse transcribed with the High Capacity cDNA Reverse Transcription Kit (Applied Biosystems). Quantitative real-time PCR was performed using an iCycler thermal cycler and iQ SYBR Green Supermix (Bio-Rad). Levels of ΔNp73 mRNA were assessed using the following primers: 5′- TGTACGTCGGTGACCCCGCACG-3′ and 5′-TCGGTGTTGGAGGGGATGACA-3′. Data are presented as average ± SD.

### Statistical Analysis

Statistical analysis was performed using the Student’s *t*-test. Results are expressed as averages ± SE, if not specifically indicated. Results were considered significant at values of *p*<0.05.

## Results

### ΔNp73 Protein is Upregulated in Patients with GERD

**Figure 1:**
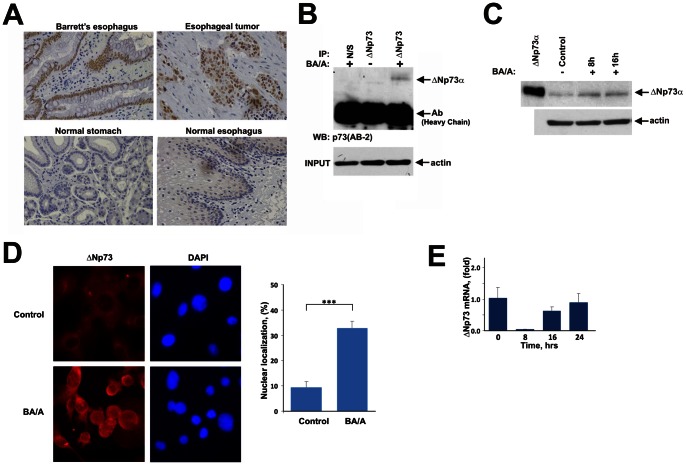
Gastroesophageal reflux causes accumulation of the ΔNp73 protein. Exposure of esophageal cells to bile acids at low pH leads to accumulation of the ΔNp73 protein and its translocation to the nucleus. **A.** Representative immunohistochemical staining for ΔNp73 in the human Barrett’s esophagus (top left panel), esophageal tumor (top right panel), normal stomach and esophagus (bottom panels). Patients with Barrett’s metaplasia express high levels of ΔNp73 in epithelial cells whereas expression of this protein is low in the normal stomach and esophagus. Esophageal tumor specimen was used as a positive control. **B.** Endogenous ΔNp73 protein was immunoprecipitated from cellular extracts of CP-A cells 7 hours after treatment with 100 µM BA cocktail, pH 4.0 for 30 min. Non-specific rabbit antibody was used as a negative control (N/S). Input protein levels were normalized to β-actin. **C.** Western blot analysis of total cell extracts from SK-GT-4 cells stably transfected with ΔNp73α plasmid. Cells were collected at the indicated time after treatment with 100 µM BA cocktail, pH 4.0 for 30 min. **D.** Representative immunofluorescent staining for the ΔNp73 protein using an anti-FLAG tag antibody in stably transfected SK-GT-4 cells before and after BA/A treatment. Cells were fixed 7 hours after treatment with 100 µM BA cocktail, pH4.0 for 30 min. Graph shows percentage of cells with nuclear localization of ΔNp73α protein (***p<0.001). **E.** Levels of ΔNp73 mRNA were not increased following BA/A treatment. Real-time PCR analysis was performed in CP-A cells at the indicated time after treatment with BA/A (100 µM, 30 min).

Expression of ΔNp73 was not previously assessed in patients with Barrett’s metaplasia. Therefore, we first analyzed the expression of ΔNp73 protein in patients with BE who were also diagnosed with GERD. Sixteen BE biopsies, 5 normal esophageal and 7 gastric specimens were analyzed by immunohistochemistry with ΔNp73-specific antibody. Since GERD-associated esophageal tumors overexpress ΔNp73 [10], specimens of esophageal adenocarcinoma with high levels of ΔNp73 protein were used as a positive control (Figure 1A, top right panel). In the normal esophagus and stomach, ΔNp73 immunoreactivity was weak and primarily found in the cytoplasm of epithelial cells (Figure 1A, bottom panels). In contrast, nuclear ΔNp73 staining was increased in all specimens of Barrett’s esophagus; moderate to strong nuclear staining (+2 and +3) was found in epithelial cells in 5 out of 16 (31%) cases (Figure 1A, top left panel). Some specimens also showed an increased cytoplasmic staining. Thus, induction of oncogenic isoforms of p73, ΔNp73, is an early event in the multistep tumorigenic process in the esophagus.

### Treatment of Esophageal Cells with Bile Acids/Acid Induces ΔNp73 Protein

To investigate how ΔNp73 is regulated by gastroesophageal reflux, these conditions were recapitulated *in vitro* using previously published measurements of pH, concentration and composition of bile acids in the refluxate of GERD patients [18]. To mimic a typical episode of gastroesophageal reflux, CP-A Barrett’s esophageal cells were treated with a single dose of 100 µM bile acid cocktail, pH 4.0 for 30 min, and the ΔNp73 protein was analyzed by Western blotting following the immunoprecipitation with a ΔNp73-specific antibody. The immunoprecipitation was used to improve specificity of our analysis. We found that exposure of esophageal cells to bile acids in acidic conditions (BA/A) leads to strong upregulation of endogenous ΔNp73α protein, similar to that found in BE patients (Figure 1B). The BA/A treatment also resulted in an increase of exogenous ΔNp73α protein in SK-GT-4 esophageal cells, in which detection of ΔNp73 was facilitated by stable transfection of FLAG-tagged ΔNp73 (Figure 1C). Notably, the ΔNp73 protein was translocated to the nuclei of SK-GT-4 cells treated with BA/A, although an increased cytoplasmic and perinuclear staining was also observed (Figure 1D). Nuclear ΔNp73 protein was found in approximately 30% cells. Next, we asked whether mRNA levels of ΔNp73 are changed following treatment of CP-A cells with BA/A. We found that ΔNp73 mRNA was not increased in BA/A-treated CP-A cells (Figure 1E), and a similar effect was seen in other esophageal cell lines BAR-T1, SK-GT-4 and Flo-1 (data not shown) indicating that ΔNp73 protein is regulated by posttranslational mechanisms.

### c-Abl, IKK and p38 Kinases Regulate the ΔNp73 Protein in BA/A-treated Cells

**Figure 2:**
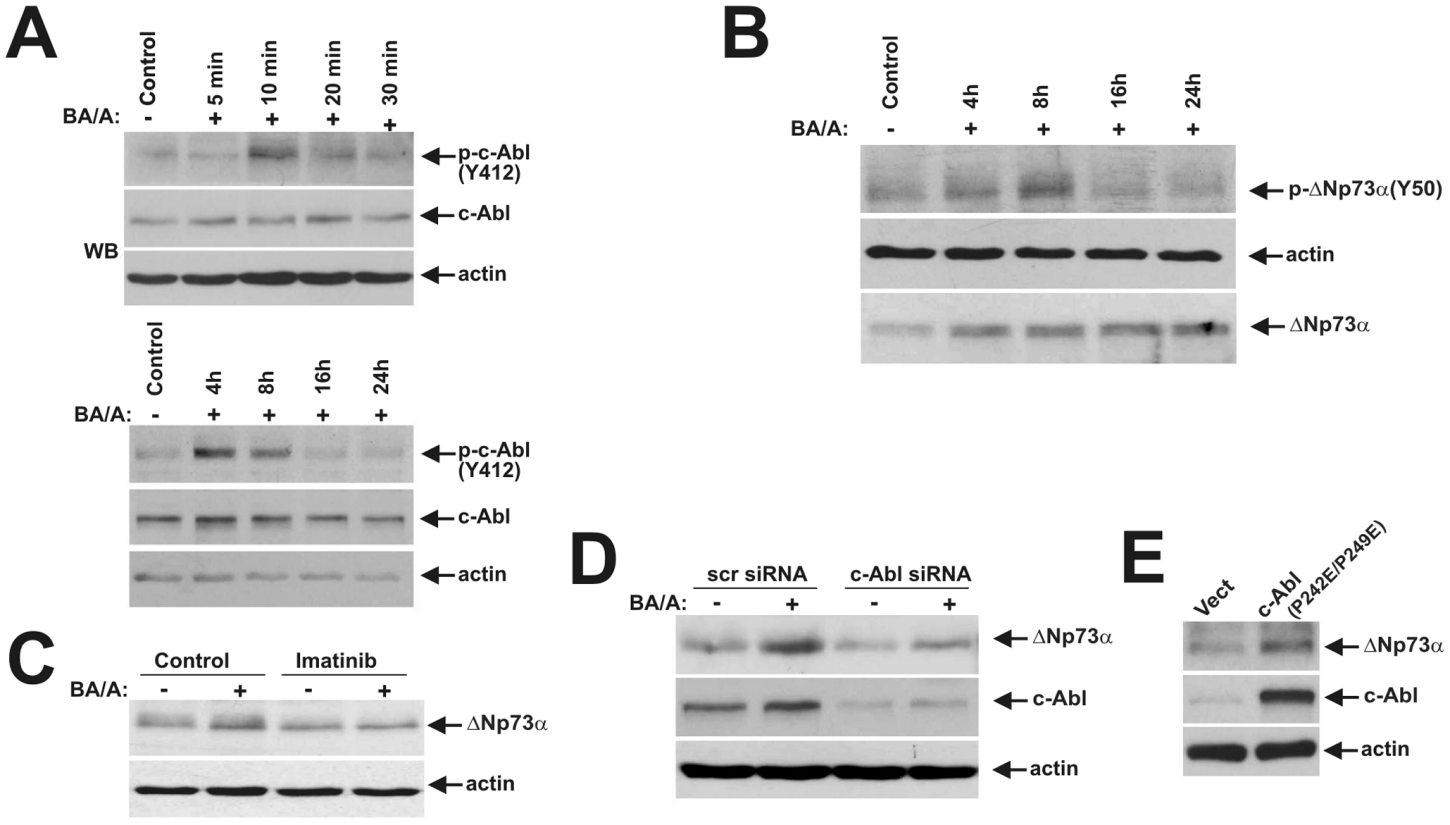
Exposure of esophageal cells to BA/A leads to activation of c-Abl kinase and upregulation of ΔNp73 protein. **A.** Dynamics of c-Abl phosphorylation in BA/A-treated cells. SK-GT-4 cells were treated with 100 µM BA cocktail, pH 4.0. Extracts were collected at the indicated time and analyzed by Western blotting. Early (5–30 minutes) and late (4–24 hours) time points are shown. **B.** Western blot analysis for phospho-ΔNp73(Y50) in SK-GT-4 cells stably transfected with ΔNp73α. Cells were collected at indicated time after treatment with BA/A (100 µM, 30 min). **C.** c-Abl chemical inhibitor Imatinib downregulates the ΔNp73 protein. SK-GT-4 cells were pretreated with Imatinib (30 µM) for 1 hour, treated with BA/A (100 µM) for 30 minutes and then incubated for an additional 7 hours in the presence of the indicated inhibitor. Total cell extracts were analyzed by Western blotting. **D.** Inhibition of c-Abl by siRNA leads to downregulation of the ΔNp73 protein in BA/A-treated cells. SK-GT-4 cells were transfected with c-Abl-specific siRNA for 48 hours, treated with BA/A (100 µM) for 30 minutes, and then collected 7 hours after treatment. Levels of ΔNp73 were analyzed by Western blotting. **E.** Transfection of constitutively active c-Abl leads to upregulation of the ΔNp73 protein. SK-GT-4 cells were transfected with the c-Abl (P242E/P249E)-pcDNA3 vector for 24 hours and analyzed by Western blotting.

Since protein stabilization via phosphorylation has been demonstrated for all members of the p53 family, including p73, we first assessed the phosphorylation of ΔNp73 protein after BA/A treatment using pan-phosphoserine and pan-phosphotyrosine antibodies. Our analyses revealed that BA/A treatment increases phosphorylation of the ΔNp73 protein at tyrosine and serine residues suggesting that phosphorylation may play a role in the regulation of ΔNp73 (Figure S1). These findings led us to the investigation of c-Abl non-receptor tyrosine kinase. We found that treatment with BA/A leads to an increased phosphorylation of c-Abl kinase at Tyr412 (Figure 2A). Interestingly, c-Abl is phosphorylated in a bimodal fashion with two peaks at early (5–10 min) and later time points. The peak phosphorylation of c-Abl was observed 4–8 hours after BA/A treatment and then gradually decreased. To analyze whether c-Abl kinase is involved in the regulation of ΔNp73 protein, cell extracts from BA/A treated and control SK-GT-4 cells were analyzed for ΔNp73(Y50) phosphorylation using a phospho-specific antibody, which recognizes the epitope of ΔNp73 protein phosphorylated by c-Abl. Our analyses found that treatment with BA/A leads to an increased phosphorylation of ΔNp73 at Tyr50 (Figure 2B). BA/A treatment also increased ΔNp73(Y50) phosphorylation in CP-A and BAR-T1 cells (data not shown). To investigate further the role of c-Abl, we inhibited this kinase with chemotherapeutic agent STI571/Imatinib (30 µM). Inhibition of c-Abl with Imatinib effectively suppressed the induction of ΔNp73 by BA/A (Figure 2C). c-Abl kinase was next downregulated with specific siRNA in SK-GT-4 cells, which were then treated with BA/A (Figure 2D). We found that c-Abl-deficient cells lost the ability to induce ΔNp73, demonstrating that c-Abl kinase regulates ΔNp73 protein in GERD conditions. Indeed, when SK-GT-4 cells were transfected with a constituvely active c-Abl (P242E/P249E) mutant, this caused an upregulation of ΔNp73 protein (Figure 2E).

**Figure 3:**
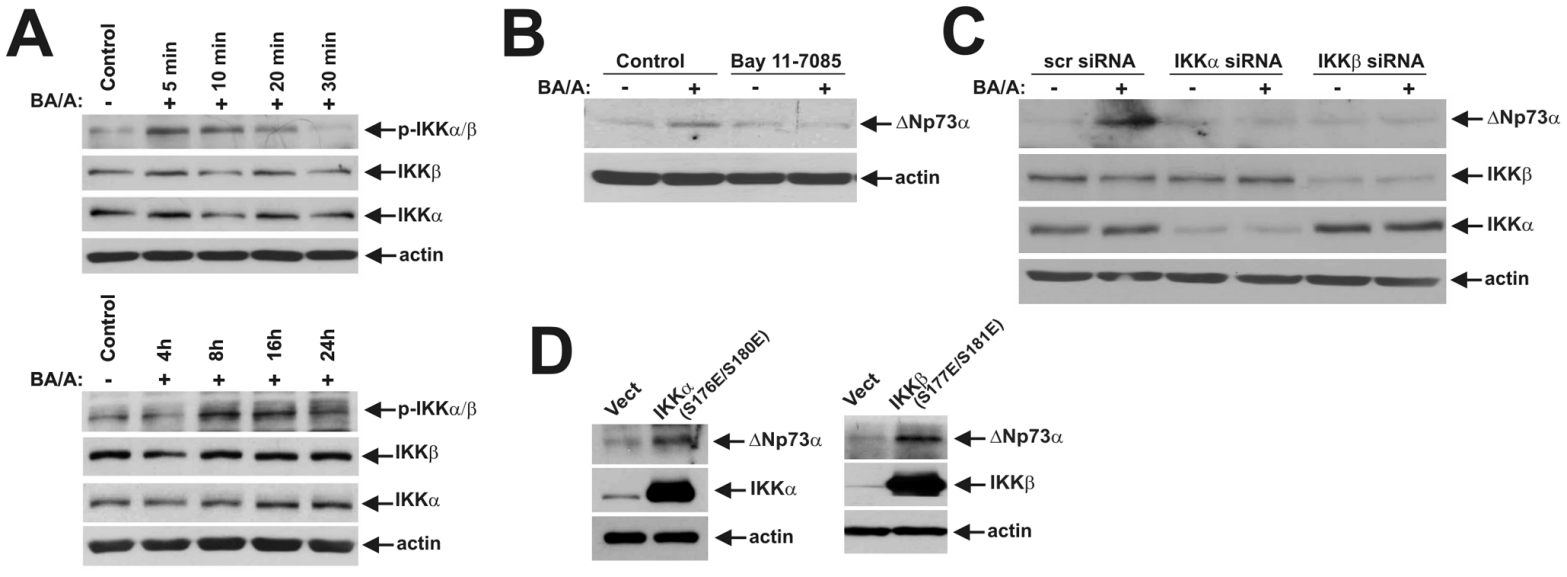
Exposure of esophageal cells to BA/A leads to activation of IKKα/β kinases and upregulation of the ΔNp73 protein. **A.** Dynamics of IKKα/β phosphorylation in BA/A-treated cells. SK-GT-4 cells were treated with 100 µM BA cocktail, pH 4.0. Extracts were collected at the indicated time points and analyzed by Western blotting. Early (5–30 minutes) and late (4–24 hours) time points are shown. **B.** IKK kinase inhibitor Bay11–7085 downregulates the ΔNp73 protein. SK-GT-4 cells were pretreated with Bay11–7085 (10 µM) for 1 hour, treated with BA/A (100 µM) for 30 minutes and then incubated for an additional 7 hours in the presence of the indicated inhibitor. Total cell extracts were analyzed by Western blotting. **C.** Inhibition of IKK kinases by siRNA leads to downregulation of the ΔNp73 protein in BA/A-treated cells. SK-GT-4 cells were transfected with IKKα- or IKKβ- specific siRNA for 48 hours, treated with BA/A (100 µM) for 30 minutes, and then collected 7 hours after treatment. Levels of ΔNp73 were analyzed by Western blotting. **D.** Transfection of constitutively active IKKα and IKKβ mutants leads to upregulation of the ΔNp73 protein. SK-GT-4 cells were transfected with the indicated mutants for 24 hours and analyzed by Western blotting.

**Figure 4:**
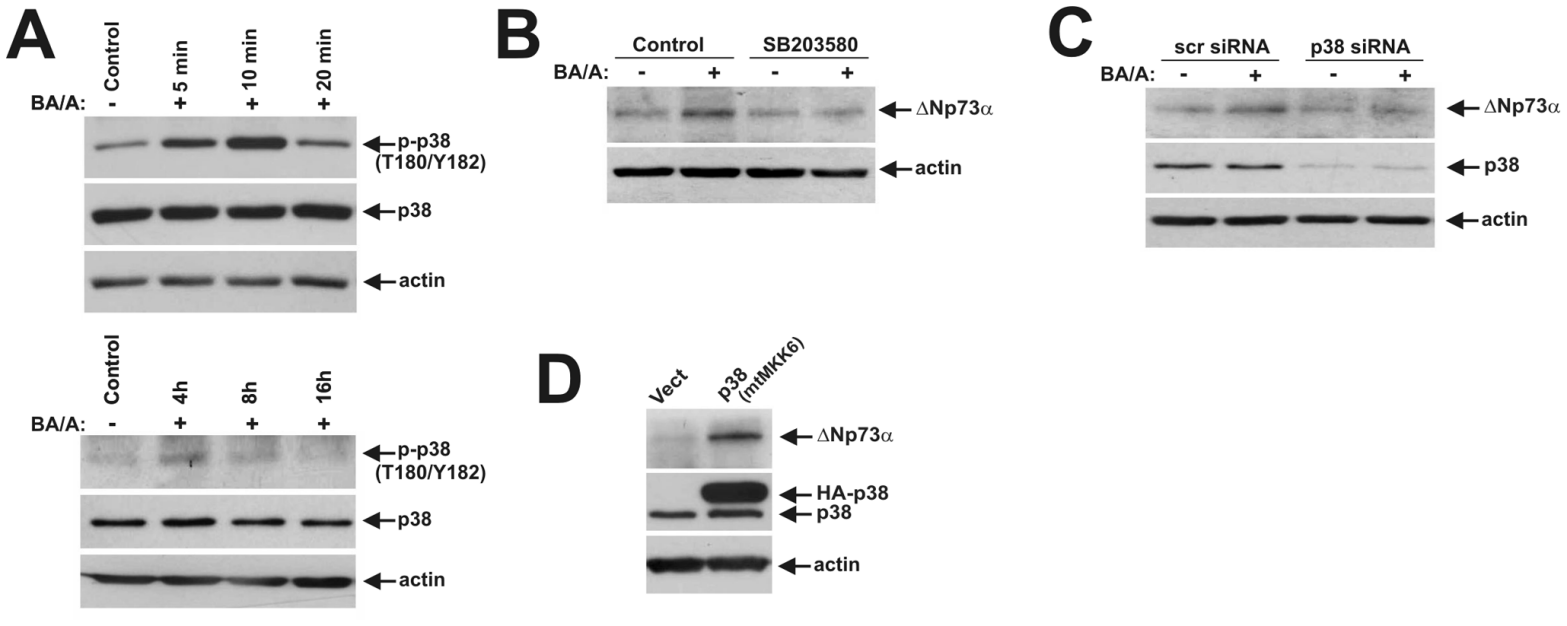
Exposure of esophageal cells to BA/A leads to activation of p38 MAPK kinase and upregulation of ΔNp73. **A.** Dynamics of p38 protein phosphorylation in BA/A-treated cells. SK-GT-4 cells were treated with 100 µM BA cocktail, pH 4.0. Extracts were collected at the indicated time and analyzed by Western blotting. Early (5–30 minutes) and late (4–24 hours) time points are shown. **B.** p38 MAPK kinase inhibitor SB203580 downregulates the ΔNp73 protein. SK-GT-4 cells were pretreated with SB203580 inhibitor (10 µM) for 1 hour, treated with BA/A (100 µM) for 30 minutes and then incubated for an additional 7 hours in the presence of the inhibitor. Total cell extracts were analyzed by Western blotting. **C.** Downregulation of p38 MAPK by siRNA leads to inhibition of the ΔNp73 protein in BA/A-treated cells. SK-GT-4 cells were transfected with p38-specific siRNA for 48 hours, treated with BA/A (100 µM) for 30 minutes, and collected 7 hours after treatment. Protein levels of ΔNp73 were analyzed by Western blotting. **D.** Co-transfection of p38 MAPK with MKK6 (S207E/T211E) mutant, which activates p38 kinase, leads to upregulation of the ΔNp73 protein. SK-GT-4 cells were transfected with the indicated constructs for 24 hours and then analyzed by Western blotting.

**Figure 5:**
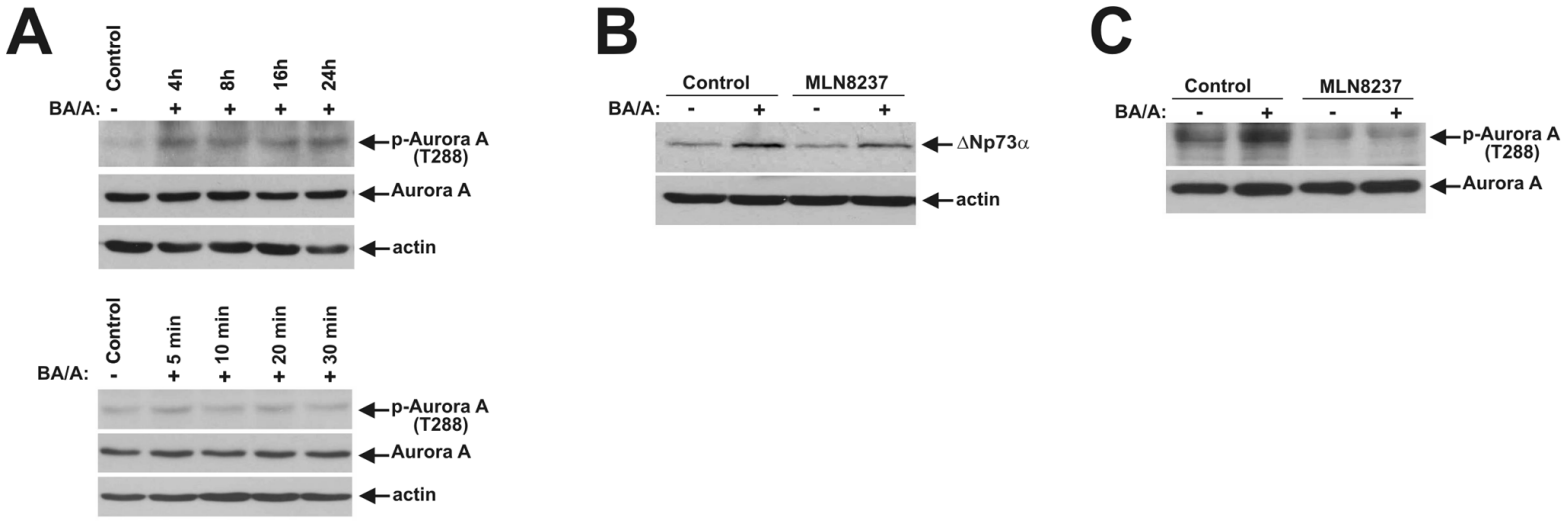
Exposure of esophageal cells to BA/A activates Aurora A kinase. **A.** Dynamics of Aurora A phosphorylation in BA/A-treated cells. SK-GT-4 cells were treated with 100 µM BA cockail, pH 4.0. Extracts were collected at the indicated time points and analyzed by Western blotting. Early (5–30 minutes) and late (4–24 hours) time points are shown. **B.** Chemical inhibitor of Aurora A MLN8237 does not affect the ΔNp73 protein. SK-GT-4 cells were pretreated with MLN8237 (0.5 µM) for 1 hour, treated with BA/A (100 µM) for 30 minutes and then incubated for an additional 7 hours in the presence of the indicated inhibitor. Total cell extracts were analyzed by Western blotting. **C.** Aurora A kinase inhibitor MLN8237 efficiently blocked autophoshorylation of Aurora A kinase.

Given that bile acids treatment can induce both cellular stress and proliferation, we also examined corresponding serine-threonine kinases p38 MAPK, IKKα/β and Aurora A [8], [19], [20]. As a prelude to our analyses, regulation of these kinases was assessed in SK-GT-4 cells treated with BA/A. We found that BA/A treatment leads to phosphorylation of IKKα at Ser176/180, IKKβ at Ser177/181, p38 at T180/Y182, and Aurora A at Thr288, which is indicative of their activation. Similar to c-Abl, the phosphorylation of IKK kinases occurred in a bimodal manner (Figure 3A). In contrast, phosphorylation of p38(T180/Y182) occurred shortly after treatment with a maximum at 10 minutes and then rapidly decreased (Figure 4A), while Aurora A phosphorylation remained steady (Figure 5A). Induction of ΔNp73 was then analyzed in cells treated with chemical inhibitors at concentrations previously reported to be effective for inhibition of the corresponding kinases. Bay 11–7085 (10 µM) and SB203580 (10 µM), which inhibit IKK and p38 kinases, respectively, strongly inhibited ΔNp73 protein, whereas Aurora A inhibitor MLN8237 (0.5 µM) did not have a significant effect (Figures 3B, 4B, 5B). To verify the inhibition of Aurora A kinase, we analyzed its auto-phosphorylation and found that MLN8237 efficiently suppressed the Aurora A activity (Figure 5C).

To corroborate the role of IKKα/β and p38 kinases, these proteins were downregulated with specific siRNAs in SK-GT-4 cells, which were then treated with BA/A (Figures 3C, 4C). We found that cells deficient in either IKKα/β or p38 were unable to induce ΔNp73, demonstrating that these kinases are involved in the regulation of the ΔNp73 protein in GERD conditions. These findings were further confirmed by transfection of constituvely active IKKα (S176E/S180E) and IKKβ(S177E/S181E) mutants, and co-transfection of p38 with MKK6 (S207E/T211E), which activates p38 (Figures 3D, 4D). Combined, these data show that c-Abl, p38 and IKK kinases regulate the ΔNp73 protein in BA/A-treated cells.

### Cytokines IL-1β and TNFα Regulate the ΔNp73 Protein

**Figure 6:**
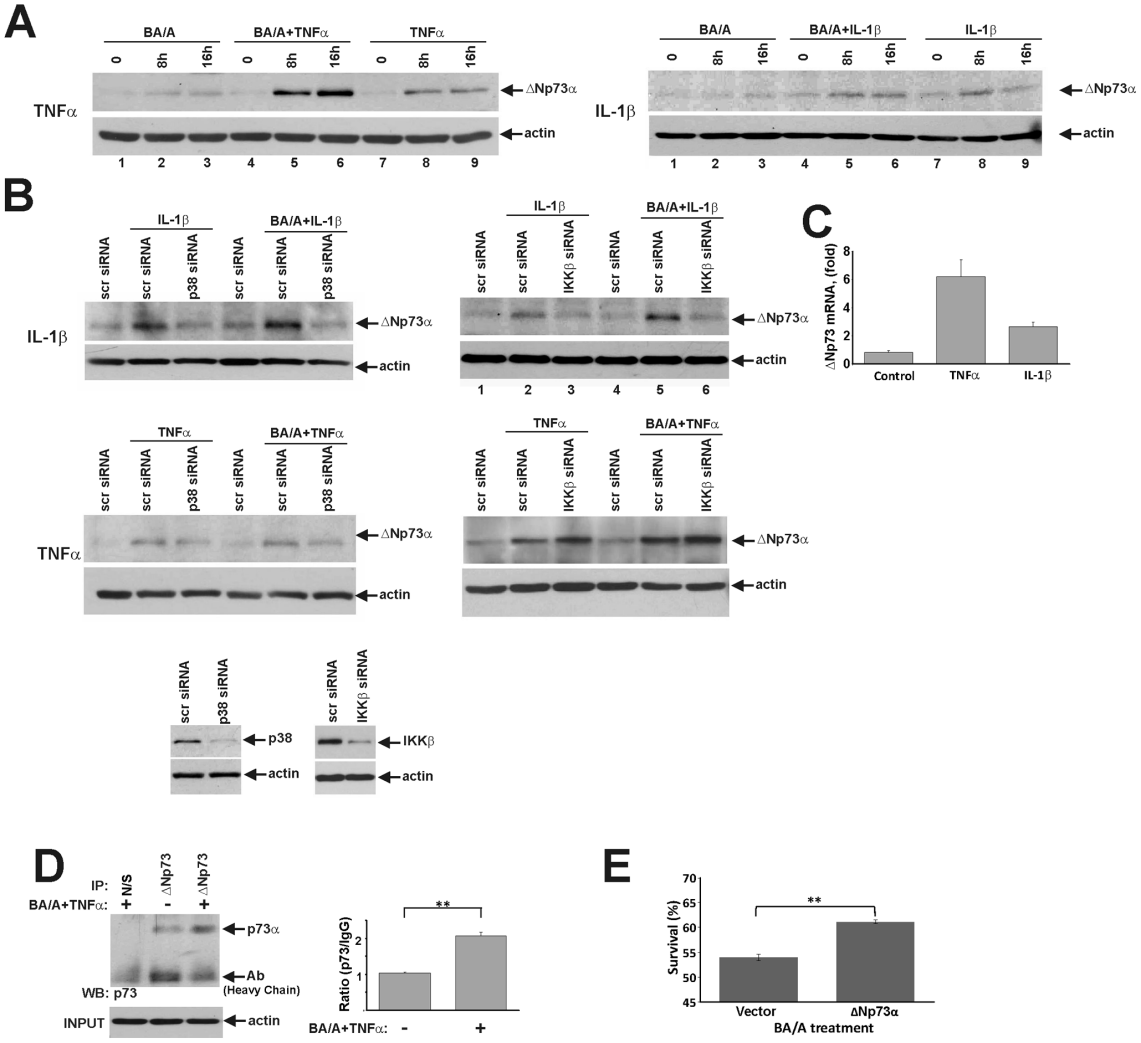
Pro-inflammatory cytokines IL-1β and TNFα induce accumulation of ΔNp73 protein. **A.** SK-GT-4 cells were treated with either BA/A (100 µM, 30 min) or cytokines (IL-1β, TNFα) or their combinations and then analyzed at the indicated time points. A combined treatment with BA/A and cytokines further enhances induction of ΔNp73 protein. **B.** Western blot analysis of ΔNp73 in SK-GT-4 cells treated as in A in the presence of siRNAs against p38 or IKKβ kinases. Bottom panel: downregulation of p38 and IKKβ kinases by siRNA is shown. **C.** Real-time PCR analysis of ΔNp73 mRNA was performed 8 hours after treatment with either IL-1β (20 ng/ml) or TNFα (20 ng/ml) in CP-A cells. **D.** BAR-T1 cells were treated with BA/A (100 µM) and TNFα (20 ng/ml). Cellular extracts were collected from treated and untreated cells. The endogenous ΔNp73 protein was immunoprecipitated using the ΔNp73 (N-16) antibody. The ΔNp73-p73 binding was analyzed by Western blotting with p73 antibody (Bethyl), which specifically recognizes p73 protein and does not cross-react with ΔNp73. Non-specific goat antibody was used as a negative control (N/S). Input protein levels were normalized to β-actin. Bottom panel: Relative binding of ΔNp73 to p73 was measured by densitometry; p73/IgG ratio is shown (**p<0.01). ΔNp73-p73 binding in untreated cells was arbitrarily set at 1. **E.** Overexpression of ΔNp73α significantly increases (**p<0.01) the survival of SK-GT-4 cells exposed to BA/A. SK-GT-4 cells, stably transfected with either ΔNp73α (ΔNp73) or empty (Vector) plasmid, were treated with BA/A (100 µM) for 20 min and cell survival was analyzed using MTT assay 24 hours after BA/A treatment.

Considering that p38 and IKKβ are well-known mediators of inflammatory response and that inflammation plays a critical role in esophageal tumorigenesis, we explored their role in more detail. To induce these kinases, SK-GT-4 cells were treated with pro-inflammatory cytokines IL-1β (20 ng/ml) or TNFα (20 ng/ml) alone or in combination with BA/A and then analyzed for ΔNp73 protein expression at the indicated time points. Protein levels of ΔNp73 were strongly increased following treatment with both cytokines (Figure 6A). Moreover, IL-1β and TNFα synergized with BA/A in inducing ΔNp73 (Figure 6A, compare lanes 3 with 6 and 9). To assess the role of the kinases, ΔNp73 levels were analyzed in cytokine-treated cells, in which p38 or IKKβ were downregulated with specific siRNAs. We found that downregulation of p38 results in inhibition of ΔNp73. Interestingly, downregulation of IKKβ was only effective in IL-1β-treated cells, while no effect was observed in cells treated with TNFα (Figure 6B), suggesting that additional mechanisms may play a role in the regulation of ΔNp73. To address this question, we assessed whether cytokines affect levels of ΔNp73 mRNA. Our analysis revealed that cytokines induce transcription of ΔNp73 in CP-A cells (Figure 6C) and similar induction was found in other esophageal cell lines (HET-1, Flo-1; data not shown). This implies that IL-1β and TNFα regulate ΔNp73 by dual mechanisms, i.e. phosphorylation of ΔNp73 protein and induction of ΔNp73 transcription. Next, we asked whether upregulation of endogenous ΔNp73 leads to an increased binding to p73, which has been demonstrated to inhibit p73 activity [21]. Barrett’s esophageal cell line BAR-T1 was treated with BA/A and TNFα (20 ng/ml), as described above, and p73-ΔNp73 binding was analyzed by co-immunoprecipitation using ΔNp73-specific antibody (Figure 6D). Our analysis revealed that BA/A and TNFα treatment increases not only levels of ΔNp73 but also its interaction with p73. Next, we determined whether the ΔNp73α protein increases the survival of SK-GT-4 cells treated with BA/A. We found that survival of SK-GT-4 cells overexpressing ΔNp73 was significantly higher than control cells transfected with an empty vector (Figure 6E).

## Discussion/Conclusion

In the present study, we investigated the regulation of oncogenic isoform of p73, ΔNp73, in precancerous conditions associated with gastroesophageal reflux and Barrett’s esophagus, which are known risk factors for the development of esophageal adenocarcinoma. We found that the ΔNp73 protein is strongly induced in Barrett’s epithelium of GERD patients, where this protein was primarily localized in the nuclei of epithelial cells. Approximately 30% of patients with Barrett’s metaplasia were found to have an increased expression of the ΔNp73 protein.

We and others have previously reported that ΔNp73 functions as an oncogenic protein immortalizing primary murine cells and cooperating with other cellular oncogene in tumorigenic transformation [11], [12]. These findings were further supported by animal studies showing that isoform-specific deletion of ΔNp73 impairs tumor formation in mice [22]. The oncogenic potential of ΔNp73α is plausibly attributed to its inhibitory interactions with p53 and p73 tumor suppressors. ΔNp73α functions as a dominant-negative inhibitor of transcriptional activities of p53 and p73. It has been found that ΔNp73α inhibits multiple p73 and p53 transcriptional targets, including ones that are involved in induction of apoptosis and cell cycle arrest. Mechanistically, inhibition of p73 and p53 appears to be determined by the stoichiometric ratio between ΔNp73α and p53/p73 molecules [6]. Our data suggest that gastroesophageal reflux may shift this ratio toward ΔNp73. Inflammation, in particular, may have a strong effect through its influence on the ΔNp73 protein levels. We found an increased binding of p73 protein to ΔNp73 in BE cells treated with BA/A and TNFα, which has been previously demonstrated to inhibit the p73 activity [21]. ΔNp73α also increased survival of esophageal cells exposed to BA/A.

We were able to recapitulate the induction of ΔNp73 in Barrett’s esophageal cells *in vitro*. A short exposure of Barrett’s cells to acid and bile acids, which mimics a typical episode of gastroesophageal reflux, was found to induce the ΔNp73 protein and its translocation to the nucleus. The induction of ΔNp73 is mediated by posttranslational mechanisms through phosphorylation of ΔNp73 protein. Protein stabilization via phosphorylation is a common trait for all members of the p53 family. However, even for the most studied member of the family, p53, many questions remain about regulation of its protein stability [23]. What’s clear is that p53 and other members of the p53 family are phosphorylated by a large number of protein kinases at multiple amino acid residues. These phosphorylation events are interdependent, such that one or more protein modifications can nucleate subsequent events [23]. The induced pattern of protein modifications appears to be dependent on the nature of the inducing agent and cellular context. These events consequently halt interactions with the protein-degradation machinery causing protein accumulation.

We identified p38 MAPK, IKKs and c-Abl kinases to be activated by BA/A and are involved in the regulation of ΔNp73 protein. Specifically, the ΔNp73 protein is phosphorylated at Tyr50 residue by c-Abl kinase. Identification of other phosphorylation sites requires additional studies and is currently precluded by a lack of phospho-specific antibodies.

Our findings are consistent with previous studies showing that activation of p38 kinase contributes to an increase in proliferation and decrease in the apoptosis rate that were found in esophageal metaplastic cells exposed to reflux [24]. Importantly, activation of p38 and IKK kinases has been found not only *in vitro* but also in the Barrett’s epithelium following reflux *in vivo* [24], [25]. In controlled studies of patients with BE, acid perfusion significantly activated p38 kinase in the metaplastic mucosa but not in normal esophageal epithelium [24].

We found that inhibition of either p38, IKK or c-Abl kinases is sufficient to suppress ΔNp73 protein, suggesting that the combined phosphorylation at various serine/threonine/tyrosine residues are required for full stabilization of ΔNp73 protein. In addition, previous studies have demonstrated multiple functional interactions between p38, IKK and c-Abl kinases. c-Abl has been found to activate the p38 signaling pathway [26]. p38, in turn, can affect phosphorylation and activity of IKK kinases [27], [28]. As a result, suppression of either kinase inhibits ΔNp73.

On a practical note, our studies imply that inhibition of upstream regulatory kinases by chemotherapeutic agents may be used as a plausible strategy for inhibition of ΔNp73 protein. Among them, c-Abl inhibitor Imatinib, which is employed for treatment of chronic myeloid leukemia, was found to efficiently inhibit ΔNp73 in esophageal cells exposed to BA/A.

We also analyzed Aurora A kinase, which has been found to phosphorylate and induce p73 protein [29]. Although the phosphorylation sites for Aurora A are retained in the ΔNp73 molecule, we were unable to demonstrate that inhibition of this kinase has a significant effect on ΔNp73 protein levels in BA/A-treated cells.

Another important finding directly related to the mechanism of ΔNp73 regulation is the role of cytokines. Pro-inflammatory cytokines, such as IL-1β and TNFα, are induced by reflux damage and contribute to esophageal tumorigenesis [30]. Tissue-specific overexpression of IL-1β induces esophageal neoplasias in mice [4]. We found that IL-1β and TNFα are strong inducers of ΔNp73. These cytokines further enhance induction of the ΔNp73 protein by BA/A. Two kinases, p38 and IKKβ, are involved in this regulatory mechanism as their inhibition affects induction of ΔNp73 by cytokines. The latter kinase has been recently reported to directly phosphorylate ΔNp73 [31]. We also found that cytokines upregulate ΔNp73 mRNA. Further studies are needed to investigate this mechanism. Combined, our studies suggest that induction of ΔNp73 protein by gastroesophageal reflux may contribute to tumorigenic transformation of Barrett’s metaplasia, especially in conditions of chronic inflammation, when BE metaplastic cells are constantly exposed to pro-inflammatory cytokines. Two factors regulate levels of the ΔNp73 protein in GERD conditions: direct exposure of esophageal epithelial cells to bile acids/acid and paracrine effect of immune cells mediated by pro-inflammatory cytokines IL-1β and TNFα.

## Supporting Information

Figure S1 **Phosphorylation of ΔNp73 at serine and tyrosine residues is increased after BA/A treatment.** AGS cell stably transfected with FLAG-tagged ΔNp73α plasmid were harvested 4 hours after BA/A treatment (100 µM, 30 min). The ΔNp73a protein was then immunoprecipitated with the M2-affinity gel and analyzed by Western blotting using either p-Ser or p-Tyr antibodies.
(TIF)
