# Supplementary figures and images for: Proinflammatory Cytokines and Bile Acids Upregulate ΔNp73 Protein, an Inhibitor of p53 and p73 Tumor Suppressors
Source: PLoS One. 2013 May 22;8(5):e64306. doi: 10.1371/journal.pone.0064306 (PMC3661465; doi:10.1371/journal.pone.0064306)

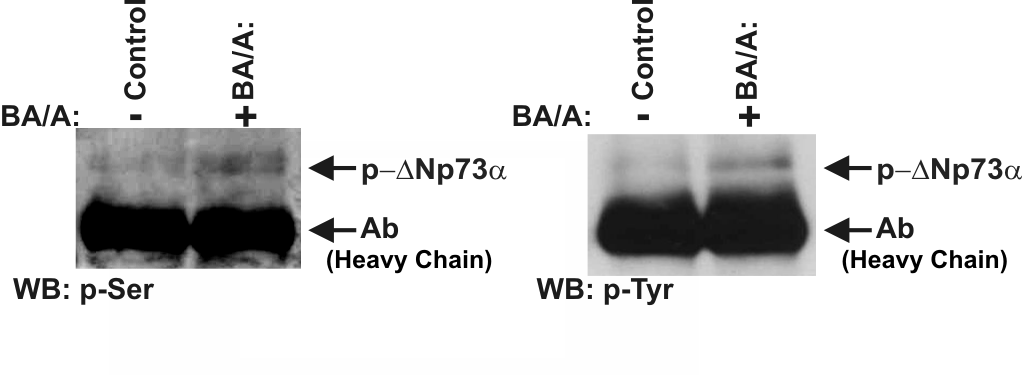

Supplement: Figure S1 — Phosphorylation of ΔNp73 at serine and tyrosine residues is increased after BA/A treatment. AGS cell stably transfected with FLAG-tagged ΔNp73α plasmid were harvested 4 hours after BA/A treatment (100 µM, 30 min). The ΔNp73a protein was then immunoprecipitated with the M2-affinity gel and analyzed by Western blotting using either p-Ser or p-Tyr antibodies. (TIF) [file pone.0064306.s001.tif]
